# Insight into Physiological and Biochemical Determinants of Salt Stress Tolerance in Tetraploid Citrus

**DOI:** 10.3390/antiox12081640

**Published:** 2023-08-19

**Authors:** Marie Bonnin, Bénédicte Favreau, Alexandre Soriano, Nathalie Leonhardt, Julie Oustric, Radia Lourkisti, Patrick Ollitrault, Raphaël Morillon, Liliane Berti, Jérémie Santini

**Affiliations:** 1 CNRS, Equipe d’Adaptation des Végétaux Aux Changements Globaux, Projet Ressources Naturelles, UMR 6134 SPE, Universite de Corse, Corte, 20250 Corsica, France; bonnin_m@univ-corse.fr (M.B.); oustric_j@univ-corse.fr (J.O.); lourkisti_r@univ-corse.fr (R.L.); berti_l@univ-corse.fr (L.B.); 2 Unité Mixte de Recherche Amélioration Génétique et Adaptation des Plantes Méditerranéennes et Tropicales (UMR AGAP) Institut, Centre de Coopération Internationale en Recherche Agronomique Pour le Développement (CIRAD), av Agropolis, 34000 Montpellier, France; benedicte.favreau@cirad.fr (B.F.); alexandre.soriano@cirad.fr (A.S.); patrick.ollitrault@cirad.fr (P.O.); raphael.morillon@cirad.fr (R.M.); 3 CEA, CNRS, BIAM, UMR7265, Aix Marseille Université, 13108 Saint Paul-Lez-Durance, France; nathalie.leonhardt@cea.fr

**Keywords:** citrus, polyploidy, salt stress, antioxidant metabolism, photosynthesis, PLS-discriminant analysis

## Abstract

Citrus are classified as salt-sensitive crops. However, a large diversity has been observed regarding the trends of tolerance among citrus. In the present article, physiological and biochemical studies of salt stress tolerance were carried out according to the level of polyploidy of different citrus genotypes. We particularly investigated the impact of tetraploidy in trifoliate orange (*Poncirus trifoliata* (L.) Raf.) (PO4x) and Cleopatra mandarin (*Citrus reshni* Hort. Ex Tan.) (CL4x) on the tolerance to salt stress compared to their respective diploids (PO2x and CL2x). Physiological parameters such as gas exchange, ions contents in leaves and roots were analyzed. Roots and leaves samples were collected to measure polyphenol, malondialdehyde (MDA), ascorbate and H_2_O_2_ contents but also to measure the activities of enzymes involved in the detoxification of active oxygen species (ROS). Under control conditions, the interaction between genotype and ploidy allowed to discriminate different behavior in terms of photosynthetic and antioxidant capacities. These results were significantly altered when salt stress was applied when salt stress was applied. Contrary to the most sensitive genotype, that is to say the diploid trifoliate orange PO2x, PO4x was able to maintain photosynthetic activity under salt stress and had better antioxidant capacities. The same observation was made regarding the CL4x genotype known to be more tolerant to salt stress. Our results showed that tetraploidy may be a factor that could enhance salt stress tolerance in citrus.

## 1. Introduction

As a consequence of climate change, accentuated by local human activities, aridification leads to land degradation and biotic impoverishment. This last phenomenon has two consequences: soil erosion and a decrease in the rate of evaporation. The disappearance of the vegetation cover and the reinforcement of natural phenomena due to global warming, such as heavy rainfall and strong winds, accentuate the erosion of the soil, which is no longer held by the roots of the plants. The soil is exposed to the elements and the top layers erode. When the vegetation cover disappears, the shaded areas become scarce. This increases the rate of evaporation, which causes salts to rise to the surface. The substrate (soil) becomes saline and prevents the development of new vegetation cover. The regions affected by this threat are also those in which crops of great importance to humans, such as citrus, are widely cultivated today.

Citrus is one of the most important fruit crops in the world and is one of the most salt sensitive species [1]. It has been demonstrated that above a threshold conductivity value of 1.4 dS m^−1^, every 1 dS m^−1^ increase results in an average of 13% decrease in citrus yield, with a 50% reduction in citrus yield being observed at electric conductivity of 5 dS m^−1^ [2]. Symptoms of salt stress adverse effects in citrus include leaf injury, growth suppression and yield decline. Salt stress decreases stomatal conductance leading to reduced CO_2_ diffusion and, ultimately, decreases net photosynthesis and increases ion accumulation.

Chloride ions (Cl^−^) excluders are usually identified as salt tolerant citrus rootstocks [3]. There is a large diversity of sensitivity in citrus toward salt stress. Few correlations exist in the ability of different genotypes to restrict sodium vs. chloride ions [4]. When comparing the most efficient excluders [Cleopatra mandarin (CL; *C. reshni* Hort. Ex Tan.) and Rangpur lime (*C. limonia* Osbeck)] with the least efficient ones [Carrizo citrange (CC; *C. sinensis* (L.) Osb.) × *Poncirus trifoliata* (L.) Raf.) and rough lemon (*C. jambhiri* Lush.)], leaf ion accumulation among genotypes is more important for Cl^−^ than for Na^+^ [4]. Because of this wide variation of leaf ion accumulation among genotypes, it is considered that Na^+^ and Cl^−^ exclusion mechanisms are heritable traits. Breeding programs were designed to produce new salt-tolerant rootstocks. The exclusion of both Cl^−^ and Na^+^ ions was observed as a quantitative character with some progenies that segregate widely for their ability to restrict the accumulation of these ions in shoot tissues and some progenies that could overtake their parent’s performances.

Maas [4] proposed a classification of the best ion excluder genotypes among citrus. Sour orange is identified as an excellent Na^+^ excluder, having also good Cl^−^ exclusion capacity. However, because of the susceptibility of this genotype to Tristeza virus, the use of this rootstock was abandoned. Tristeza virus tolerant rootstock, like trifoliate orange (*P. trifoliata*) (PO) and its hybrids (citranges and Swingle citrumelo), are unfortunately sensitive to salinity [4]. CL mandarin (*C. reshni*) (CL) is considered as an excellent parental genotype in breeding programs and leads to salt-resistant citrus hybrids. Hussain et al. [1] showed that salt stress tolerance within the *Citrus* genus was a characteristic of mandarins (*C. reshni*) and pummelos (*C. maxima* (Burm.) Merr), while all interspecific hybrids that presented good resistance to salt stress shared mandarin or pummelo as a female parent. Combava (*C. hystrix* D.C.), which belongs to the subgenus Papeda, was also classified as resistant.

Plants respond to salinity in two phases: an early osmotic phase which is fast and leads to the inhibition of the growth of young leaves, and a later phase that is slow and is associated with a change of the ion contents, which, in turn, accelerates the senescence of mature leaves [5]. The early phase begins immediately after the salt concentration at the root level reaches a critical threshold. The osmotic effects of salt at the root level leads to a reduction in leaf expansion. The late phase corresponds to the accumulation of salt at toxic doses at the level of mature leaves [4]. It is characterized by the inability to dilute the salts and senescence [5]. If the percentage of senescence of mature leaves is higher than the rate of production of new leaves, photosynthetic activity will be insufficient to provide the carbohydrate requirements of young leaves, which will lead to growth reduction [5].

Several studies carried out on citrus demonstrated that polyploid species might have better adaptive abilities than diploids (2x), particularly under salt stress [1,4,5,6]. Doubled diploids (4x) differ from their 2x parents by a doubled number of chromosomes. Tetraploid plants usually arise spontaneously by chromosome set doubling in maternal nucellar cells, which produce somatic embryos in apomictic 2x citrus genotypes [7]. Some phenotypic differences between ploidy levels include an increase in cell volume. Compared to 2x, organs of 4x have a more massive appearance. Polyploid leaves are thicker and also exhibit greater photosynthetic activity and chlorophyll content than 2x [8]. The stomata of polyploids are larger and their density is lower than in 2x. Tetraploid plants generally show stunted growth, which makes them smaller than 2x plants. Even though they have fewer but larger seeds than 2x and their fruits are larger than 2x, polyploid citrus fruits are less juicy and have a thicker rind than 2x. These differences may contribute to better adaptation capacities of 4x under salt stress conditions, especially when taking account the ability of their roots to exclude toxic ions from leaves [8]. Thus, 4x citrus genotypes, once used as rootstocks, could provide a sustainable system to cope with the toxicity problem caused by ion excess [9]. Several studies have already demonstrated the relationship between phenotypic variation in the aerial part and stress resistance in polyploid citrus [10]. In contrast, the role of the root system is poorly documented.

To study the different levels of response of citrus subjected to salt stress, 2x and 4x genotypes were selected. Hence, 2x *Poncirus trifoliata* (PO2x) and 2x Cleopatra Mandarin (CL2x) were investigated. We used the contrasted behavior of these two genotypes toward salt stress to describe the behavior of the two doubled diploid: 4x *Poncirus trifoliata* (PO4x) and 4x Cleopatra Mandarin (CL4x).

Thus, this study highlights the physiological and biochemical determinants involved in polyploids adaptation to salt stress in comparison with diploid ones. By studying both photosynthetic parameters and ROS detoxification mechanisms in leaves and roots, we characterized the adaptation of these genotypes to stress. We also examined if a better adaptation to salt stress was necessarily associated with an exclusion of the toxic ions (Cl^−^ and Na^+^) at the root level and/or if better detoxification or compartmentalization capacities of these ions at the cellular level in leaves could explain a better tolerance.

## 2. Materials and Methods

### 2.1. Plant Material

Four rootstock genotypes were selected for the comparative salt stress tolerance study. Diploid Trifoliate orange (*Poncirus trifoliata* (L.) Raf.) (Poncirus Pomeroy ICVN-0110081) (PO2x) and 2x Cleopatra mandarin (*Citrus reshni* Hort. Ex Tan) (ICVN-0110274) (CL2x) and their two doubled diploid counterparts, 4x Trifoliate orange (*Poncirus trifoliata* (L.) Raf.) (IVCN-0101116) (PO4x) and 4x Cleopatra mandarin (IVCN-0101110) (CL4x) were included (Table 1). Citrus seeds were sown between the end of December 2020 and the early beginning of January 2021. All germplasm originated from the INRAE-CIRAD collection at the San Giuliano research station in Corsica (42.286, 9.520) France. The 4x genotypes resulted from spontaneous doubling of the chromosomal stock of nucellar tissues of somatic origin and were selected by flow cytometry in seedlings of the corresponding 2x genotypes and were then transferred in the collection [7].

The trees selected for experimentation (27 plants per genotype) were distributed over two irrigation blocks in a climate-controlled greenhouse at CIRAD of Montpellier, France (43.649, 3.869). The seeds were sown in a neutral substrate (perlite). Seedlings were transplanted 4 months after germination into 3-liter pots and regular fertilization was applied [10,11]. The substrate used consisted of 1 volume of “NEUHAUS Humin-Substra N2” horticultural potting soil to 2 volumes of white peat and 3 volumes of medium-grained perlite. The trees were grown in a greenhouse under controlled conditions of temperature, humidity and natural photoperiod.

Plants selected for experimentation were grown under natural daylight with supplemental lightning, maintaining a 12 h photoperiod, by using horticultural red–blue LED projectors Alpheus Radiometrix 15 M 1006, providing a R/FR ratio about 1.2 microclimate (LED lighting complementary to solar radiation LI-180 318M0037 with 0.230047 UV %, 25.692581 B %, 19.955620 G %, 43.531086 R %, 10.590654 FR %. Alpheus, Boulevard Sellier 91230 Montgeron, France) and monitored using data-loggers (CR 1000 Campbell scientific, Campbell Park, 80 Hathern Road, Shepshed, Loughborough, Leicestershire, England, LE12 9GX) installed in each compartment. Air temperature ranging from 19 to 32 °C (day) and 16 to 18 °C (night) was measured with a PT 1000 probe under a fan-aspirated shield. Average air relative humidity (RH) ranged from 40 to 60%, and monitored with HMP45 (Vaisala, Helsinki, Finland). PPFD was measured with an SK P215 (Skye Instrument quantum sensor, Powys, UK), providing, on average, photosynthetic irradiance of 800 μmol·m^−2^·s^−1^ at the top of the canopy level during daytime. The mean photosynthetically active radiation received by the plants during their life cycle was 15.12 MJ·m^−2^·s^−1^.

The ploidy level of 2x and 4x seedlings was determined by flow cytometry (Cyflow^TM^ space Sysmex America, Inc., 577 Aptakisic Rd, Lincolnshire, IL 60069, USA) at CIRAD Montpellier with the same method described in [12]. Clonal propagation by nucellar embryogenesis was verified by genotyping using KASPar markers [13,14]. For each diploid accession, 18 heterozygous markers were selected (9 telomeric and 9 centromeric). They were developed using sequencing and previous GBS data were used to establish genetic maps of trifoliate orange and CL mandarin (our unpublished data). The results of the KASPar genotyping were analyzed via the snpclust pipeline that opens from R studio and described on the gitlab: Version 0.2 https://github.com/jframi/snpclust (accessed on 1 September 2017). Diploid and tetraploid plants with identical SNP genotype than maternal trees were selected, respectively, for PO2x/CL2x and PO4x/CL4x.

### 2.2. Salt Stress Experiment

Salt stress was conducted in climate greenhouse from 8 November to 17 December 2021. Plants selected for experimentation were about one year old. At the beginning of the experiment, PO plants were, on average, 40 to 45 cm high. CL plants were about 15–16 cm high. Genetically conforming and uniform plants were divided into two blocks: 17 stressed and 10 control plants. Vessel dimension was 11–12 cm. Watering was carried out by immersing the pots in a nutrient solution for controls and in a nutrient solution supplemented with salt for stressed plants. Watering process and frequency were chosen after testing different watering process, to finally choose the optimal way to water plants and avoid at maximum water loss. Substrate retention capacity experiment revealed that only 5% of water content was lost in 6 days. Considering these results, we decided to water the plants only once a week. The experiment revealed that 200 mL of saline solution were enough to water one vessel, using the immersion watering technique. Watering was conducted once a week, at the end of the week. Soluble Plantin^®^ fertilizer (formula 20-10-10 Oligo Element + 1.5 mg (125 g PC/L mixed solution, 5 mL/L usage) and 46% commercial urea were used. Both fertilizers were used at a concentration of 5 mL/L as nutritive solution. Stressed plants were irrigated with a nutrient solution supplemented with salt (NaCl). Control plants were irrigated with nutrient solution only. The salt concentrations supplied were steadily increased from 30 mM to 90 mM, with an increment of 20 mM per week and were then stabilized at 90 mM. We wanted to test the influence of progressive salt application to evaluate possible adaptation mechanisms. Gas exchanges were measured each time the salt concentration increased and were performed for each physiological parameter investigated. Samples for biochemistry analyses were taken at week 4 (w4:90 mM NaCl). Sampling for foliar and root chloride determinations was performed weekly. The same temperature and humidity parameters were used in the greenhouse before the experimentation and during the salt stress experiment. Air temperature ranged from 19 to 32 °C (day) and 16 to 18 °C (night). Temperature was measured with a PT 1000 probe under a fan-aspirated shield. Average air relative humidity (RH) ranged from 40 to 60%, and was monitored with HMP45 (Vaisala, Helsinki, Finland).

### 2.3. Determination of Physiological Characteristics

All the photosynthetic parameters investigated were measured on the same leaves at the beginning of the week following watering with or without salt supply. We chose fully developed leaves in the medium part of the plants. Fully developed leaves were selected with the same light exposure. Three leaves by biological replicates, at the same stage of development located in the middle part of the plants, were banded. Net photosynthetic rate (*P_net_*) and stomatal conductance (*g_s_*) were measured using a portable infrared gas analyzer LC-PRO-SD (ADC, BioScientific Ltd., Hoddeston, UK). During the experiment, photosynthetically active radiation (PAR) was applied to the leaf surface and set at 1400 μmol·m^−2^·s^−1^ [15]. The leaf temperature was set at 28 °C and the ambient carbon dioxide (CO_2_) concentration was used (390 μmol·mol^−1^). The maximum PSII quantum efficiency (*Fv/Fm*), effective PSII quantum yield (*Φ_PSII_*) and electron transport rate (ETR) were measured using an OS1p chlorophyll fluorometer (Opti-Sciences, Inc., Hudson, NH, USA). The *F_v_/F_m_* ratio was monitored on dark-adapted leaves using clips through the thylakoid membrane for 30 min [16]. For light fluorescence measurements, the fluorometer was equipped with an open clip suitable for measurements on light-adapted leaves. ΦPSII was evaluated as described by [17,18] and ETR as expressed according to Krall and Edwards (1992) [19].

### 2.4. Determination of Major Cations and Chloride Concentrations

Leaf samples were collected to estimate the content of main cations (Na^+^, K^+^, Ca^2+^, Mg^2+^, P) and Cl^−^ in leaves. Briefly, 5 g of leaves and roots (dry weight) were taken each week and dried for 24 h at 65 °C. Samples were mineralised in HNO_3_ 70% and were then diluted at 1/10. Cl^−^ concentration was determined using a chloride ion selective electrode (ISE) thermofisher scientific.

Determination of principal cations concentrations was performed using an Agilent 5100 SVDV ICP-OES with dichroic spectral combiner (DSG) technology.

### 2.5. Determination of Biochemical Characteristics

Three samples were collected for each genotype by pooling 20 leaves and/or 5 g of roots for each modality (control/stressed). Harvested samples were immediately immersed in liquid nitrogen, ground to a fine powder and stored at −80 °C.

The determination of malondialdehyde content was performed as described by [17]. About 80 mg of leaf powder was homogenized in 2 mL of 80% (*v*/*v*) ethanol. The homogenates were centrifuged at 3000× *g* at 4 °C for 10 min. Absorbance was determined at 440, 535 and 600 nm.

The hydrogen peroxide (H_2_O_2_) content was measured using the PeroxiDetect kit (Sigma Aldrich, St. Louis, MO, USA) according to the method described by [17]. The absorbance was read at 560 nm with a microplate reader (MULTISKAN FCTM, Thermo Scientific, Waltham, MA, USA). The concentration of H_2_O_2_ was determined from a standard curve.

Ascorbic acid determination was performed as described by [18]. The absorbance was read at 560 nm with a microplate reader (MULTISKAN FCTM, Thermo Scientific, Waltham, MA, USA). Total and reduced ascorbic acid contents were determined using a standard curve.

Determination of proline content was performed as described by [17]. The absorbance was read at 520 nm with a microplate reader (MULTISKAN FCTM, Thermo Scientific, Waltham, MA, USA). Proline content was determined using a standard curve.

Determination of total polyphenol content was performed according to [20]. About 500 mg of ground samples was incubated for 72 h in 10 mL of 100% methanol and was then filtered. Polyphenolic assays were performed with the “Folin Ciocalteau Phenolic Content Quantification Assay’’ kit according to Bioquochem’s instructions. Polyphenol concentrations were determined using a standard curve.

To measure the activity of enzymes involved in ROS detoxification, 54 mg of leaf powder was homogenised in 2 mL of extraction buffer (0.1 M potassium phosphate, pH 7.5) and the homogenates were centrifuged at 13,000× *g* for 30 min at 4 °C. The supernatant was collected and used for all enzyme assays and for determination of protein concentrations [17,18]. Assays for superoxide dismutase, catalase, ascorbate peroxidase and dehydroascorbate reductase were performed as described by [21]. Measurements were performed using a V-630 spectrophotometer (Jasco Inc., Tokyo, Japan).

### 2.6. Statistical Analysis

Partial least square discriminant analysis (PLS-DA) was performed to evaluate the quality of the physiological and biochemical datasets and the determination of the sample structure. Then, variable selection was assessed by sparse PLS-DA (sPLS-DA). Both statistical analyses were carried out using the mixOmics R package [22]. The tuning of the sPLS-DA was performed as recommended by the mixOmics documentation R: package version 6.1.1. 2016 https://CRAN.R-project.org/package=mixOmics http://mixomics.org/case-studies/splsda-srbct-case-study/. Because our dataset was small, the optimal number of components was determined using the Leave-One-Out (LOO) validation. Then, the optimal number of variables that was most significant was identified. Measures of variance were performed to determine if at least one diploid and/or tetraploid genotype was significantly different (at *p*-value < 0.05) than the other ones for the selected variables. Considering the low number of samples (<30), the non-parametric Kruskal–Wallis test was applied (*p* ≤ α, with α = 0.05)) as well as the size effect Eta2 which defined the magnitude of the difference between 2 groups (0.2 = small, 0.5 = medium, 0.8 = large) [23]. Pairwise comparison by Dunn’s test was then performed on these variables to measure the differences between the diploid and polyploid genotypes at *p*-value < 0.05, and the box plot was designed using the ggplot2 R package [Version 3.4.3 version 3.4.3 https://CRAN.R-project.org/package=ggplot2 (accessed on 14 August 2023) https://ggplot2.tidyverse.org]. Only sPLS-DA-selected variables are represented in Table 2 and Table 3.

## 3. Results

### 3.1. Physiological and Biochemical Parameters Explaining the Differences between the Diploid PO and CL Genotypes (PO2x and CL2x) and Their 4x Counterparts (PO4x and CL4x) under Control Conditions

**Figure 1:**
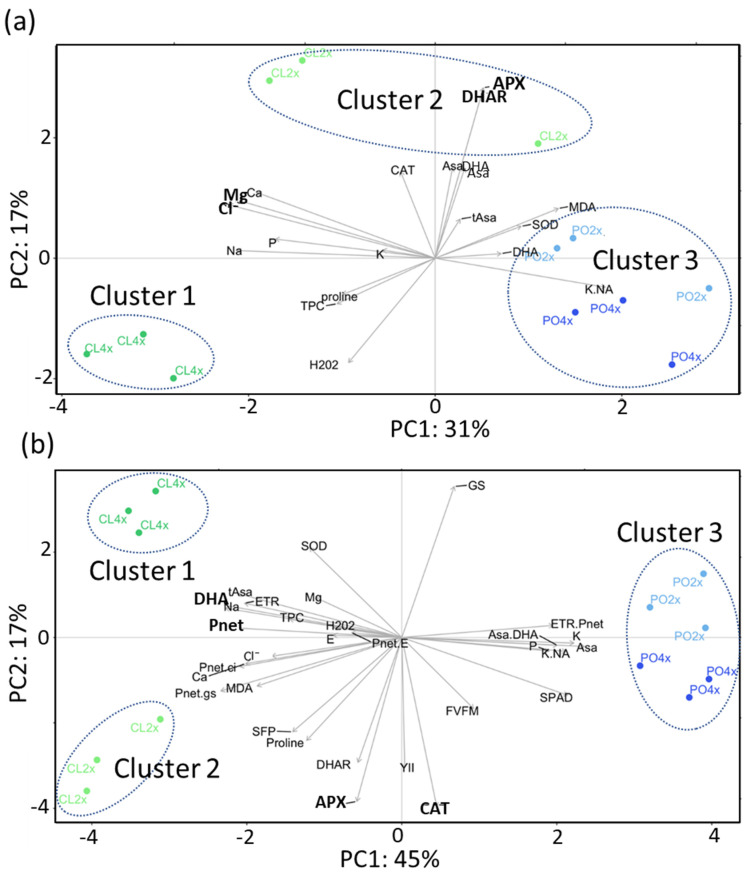
Discriminant analysis of biochemical and photosynthetic variables under control conditions. PLS-DA biplot represents discrimination of Cleopatra (CL) and trifoliate orange (PO) genotypes at two ploidy levels (2x, 4x) in roots (**a**) and leaves (**b**). Measurements were performed at W4 (90 mM). Colored dots represent biological replicates for each genotype. sPLS-DA selected variables were represented in bold.

Relevance of the variables studied to discriminate factors of the physiological and biochemical datasets from the control treatment, and structuration of the samples were assessed thanks to PLS-DA analysis (Figure 1). The first component explained 31% and 45% of the variability, for roots (Figure 1a) and leaves (Figure 1b), respectively, allowing discrimination of the two genotypes. Ploidy level was discriminated by the second component, representing 17% of the variability in both tissues, more significantly for Cleopatra (CL) than trifoliate orange (PO). All in all, in both tissues, PC1 and PC2 allowed the discrimination of three clusters: CL4x (cluster 1), CL2x (cluster 2) and PO2x/PO4x (cluster 3). For each tissue, the few variables that accounted for most of the difference between genotypes and/or ploidy were selected using sPLS-DA (Table S1; Figure 1). Significance of differences between groups was further validated for each selected variable (if *p*-value < 0.05), and the magnitude of these differences was estimated using the size effect test (Table S2).

In roots, Mg^2+^ and Cl^−^ ions content were the two most significant variables accounting for the differences between CL and PO on PC1 (Figure 1a). Cl^−^ ions content (Table S2) was significantly different between PO2x and CL2x (55.71%) and between CL4x and PO2x (58.45%). Difference between ploidy level was mainly explained by APX and DHAR for both genotypes, revealing much higher activities for the two diploids compared to the tetraploid ones. However, this difference was only significant for CL (Table S2).

In leaves, variation of *P_net_* and DHA explained the difference between the two genotypes (Figure 1b). *P_net_* was higher for CL than PO, depending on the ploidy level. PO2x had significantly the lowest *P_net_* compared to PO4x and the two CL genotypes. DHA was the variable the most contrasted between the two genotypes, since its concentration was more than 20 times higher for CL than PO. Two variables, APX and CAT, involved in the antioxidant metabolism, explained the differences between the ploidy levels for both genotypes (Figure 1b). Activities of these two enzymes were significantly higher for CL2x than CL4x, while for trifoliate orange, they were similar for PO4x than PO2x (Table S2).

### 3.2. Physiological and Biochemical Parameters Related to the Differences between the Diploid PO and CL Genotypes (PO2x and CL2x) and Their 4x Counterparts (PO4x and CL4x) under Salt Stress Conditions

**Figure 2:**
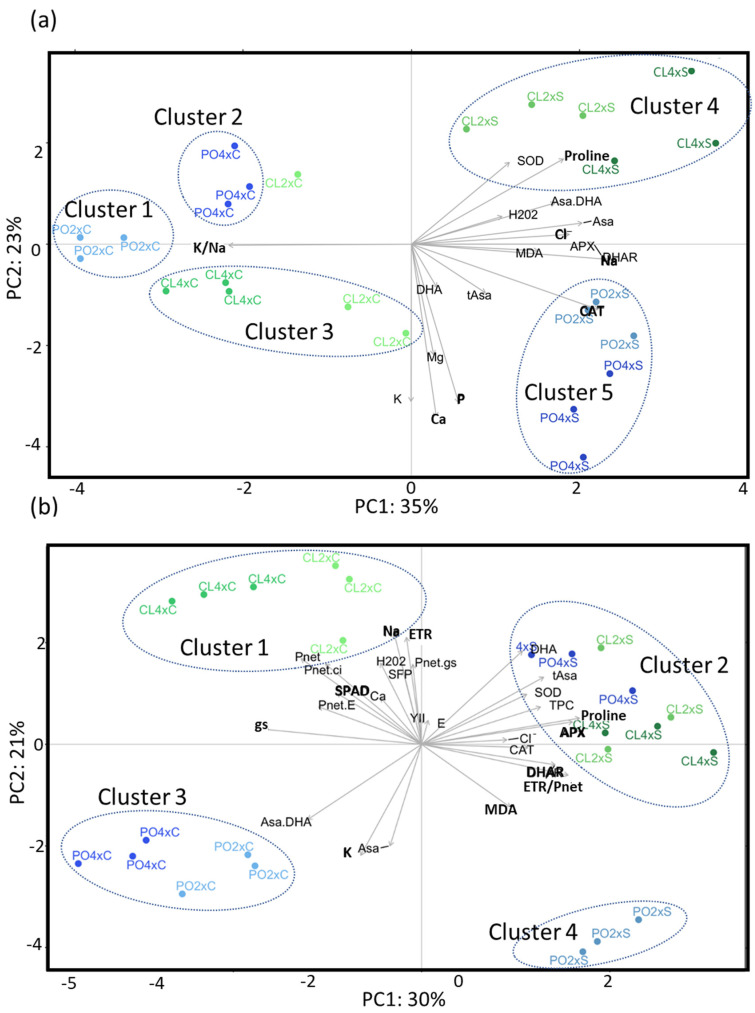
Discriminant analysis of biochemical and photosynthetic variables under control (C) and stress conditions (S). PLS-DA biplot represents discrimination of Cleopatra (CL) and trifoliate orange (PO) genotypes at two ploidy levels (2x, 4x), with (S) and without salt stress (C), in roots (**a**) and leaves (**b**). Measurements were performed at W4 (90 mM). Colored dots represent biological replicates for each genotype. sPLS-DA selected variables were represented in bold.

In a similar way to the control treatment, PLS-DA was applied on both the control and stress datasets to assess the quality and structuration of the data. The salt treatment (progressive increase in salt stress to 90 mM NaCl) explained the difference between samples (Figure 2). In both tissues, the first component PC1 was able to discriminate the control versus the stressed samples, regardless of the genotype and ploidy level. PC1 explained 35% and 30% of the variability in roots (Figure 2a) and leaves (Figure 2b), respectively. On PC2, samples were clustered in different groups formed by different genotypes and ploidy levels, according to the tissue type. PC2 explained 23% and 21% of the variability in roots and leaves, respectively.

In roots, PC2 discriminated two clusters, CL2x and 4x under salt stress (CL2xS and CL4xS) and PO4x under control conditions (PO4xC), on the top side. This component also discriminated PO2xS and PO4xs and CL2xC and CL4xC on the bottom side. Overall, the two first components underlined the increasing contrast between samples, according to genotype and ploidy level, under stress treatment compared to the control one. In root samples, 13% of the variability were still detected with PC3, explaining ploidy level discrimination for both genotypes (Figure S2).

Together, PC1 and PC2 allowed the discrimination of five clusters: PO2xC (cluster 1), PO4x (cluster 2), CL2xC/CL4xC (cluster 3), CL2xS/CL4xS (Cluster 4) and PO2xS/PO4xS (cluster 5).

In leaves, PC2 discriminated CL2xC and CL4xC, on the upper side, from PO2xC, PO4xC and, PO2xS, on the bottom side. Considering both PC1 and PC2, leaf variables allowed the clustering of four groups: CL2xC/CL4xC (cluster 1), CL2xS/CL4xS/PO4xS (cluster 2), PO2xC/PO4xC (cluster 3) and PO2xS (cluster 4). Unlike roots, ploidy level was not significantly discriminated on further components.

Overall, PLS-DA of control and stress samples, in leaves and roots, highlighted that the first factor explaining the variability of the physiological and biochemical variables was the treatment applied, and the second factor was the differences of response between genotypes.

sPLS-DA allowed selection of the most significant variables explaining the differences between samples (Table 2). In roots, seven variables were reported on the first two components (Table 2, Figure 2a), comprising five related to mineral uptake (Na^+^, Cl^−^, K^+^/Na^+^, Ca, P), and two to antioxidant metabolism (proline, CAT). Moreover, the third component was significant as well to explain the variability of the samples, with four more variables selected, all related to antioxidant metabolism: MDA, Asa, APX and DHAR (Supplementary Table S1 and Supplementary Figure S4). Biological significance of these variables was revealed thanks to pairwise comparison analysis between groups (Table 3).

**Figure 3:**
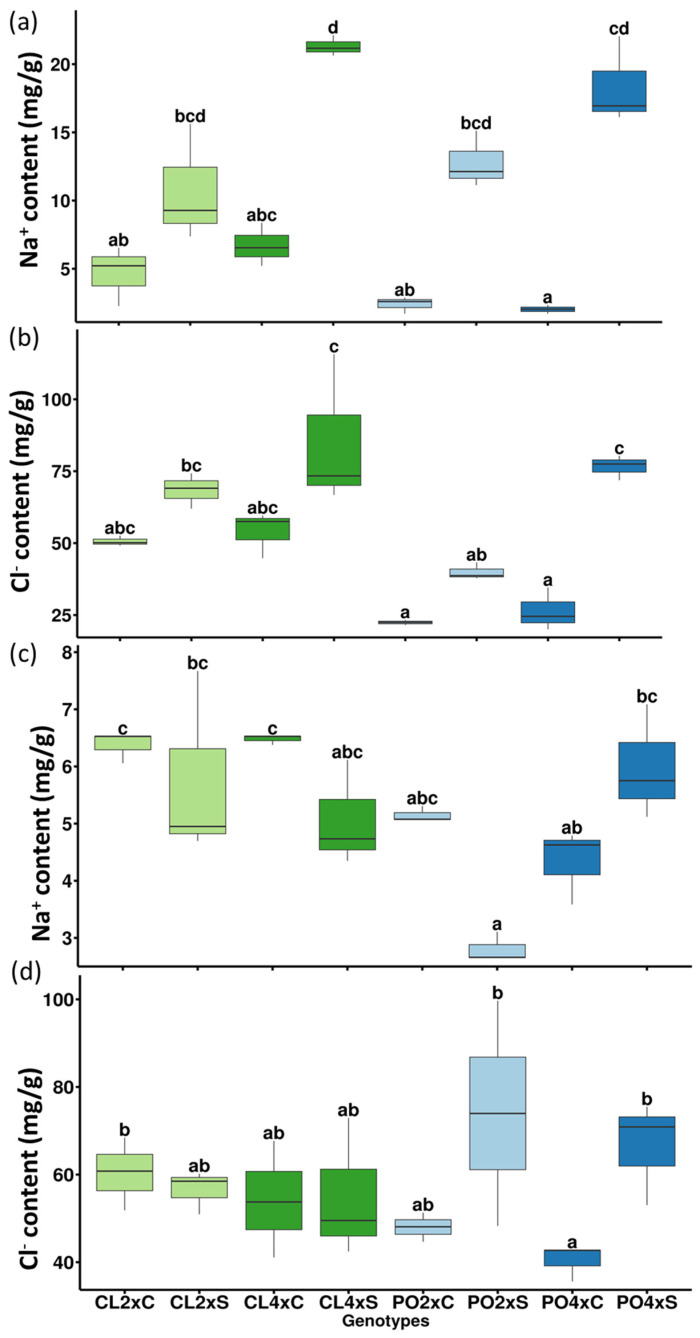
Mineral uptake. (**a**) Roots Na^+^ content and (**b**) Roots Cl^−^ content (mg/g) with (S) and without salt stress (C). Leaves Na+ and Cl^−^ content are represented in (**c**,**d**), respectively, with (S) and without salt stress (C). Measurements were performed after four weeks of salt treatment (90 mM). Data represents 3 independent measurements (*n* = 3). Significance of the values were analyzed using the Kruskal–Wallis test (*p* > 0.05) and mean comparison using Dunn’s test. Groups are separated by letters. Each group is assigned one or more letters. Groups sharing the same letter are not significantly different. Letter displays a clear and succinct way to present results of multiple comparisons.

In roots (Table 3; Figure 2a), five variables related to mineral uptake explained the differences between control and stress treatments. Na^+^ and Cl^−^ contents tended to increase for all the samples under salt stress compared to the control treatment, even though changes were not significant all the time (Table 3; Figure 3a,b). However, Na^+^ increase was significant for CL4x and PO4x only, while Cl^−^ was significant for PO4x only, when compared to their respective controls. In the most genotypes, proline content and CAT activity increased under stressed conditions. However, proline increase was significant for the CL genotypes only (Table 3), and CAT for CL4x and the two PO genotypes. Conversely, K^+^/Na^+^ ratio decreased significantly for all the stressed genotypes, compared to the control ones (Table 3). Ca^2+^ and P, the two variables on PC2, significantly increased for the PO4xS, while Ca^2+^ decreased significantly for the CL4xS only, compared to their respective control. PC3 allowed identifying two variables, APX and DHAR, explaining differences between stressed CL4x and PO2x, compared to their control. Both APX and DHAR activities increased significantly for CL4xS. CL4xC and CL4xS DHAR activities were 0.01 and 0.21 µmol·min^−1^·mg^−1^·protein, respectively, meaning an increase of 24.5-fold. Additionally, CL4xC and CL4xS APX activities were 0.02 and 0.49 µmol·min^−1^·mg^−1^·protein, corresponding to an increase of 21-fold. The same trends were reported for PO2xS compared to control ones. PO2xC and PO2xS DHAR activities were 0.02 and 0.29 µmol·min^−1^·mg^−1^·protein, while PO2xC and PO2xS APX activities were 0.05 and 0.67 µmol·min^−1^·mg^−1^·protein. This corresponds to an increase of 14.50 and 13.40-fold, respectively (Table 3).

**Figure 4:**
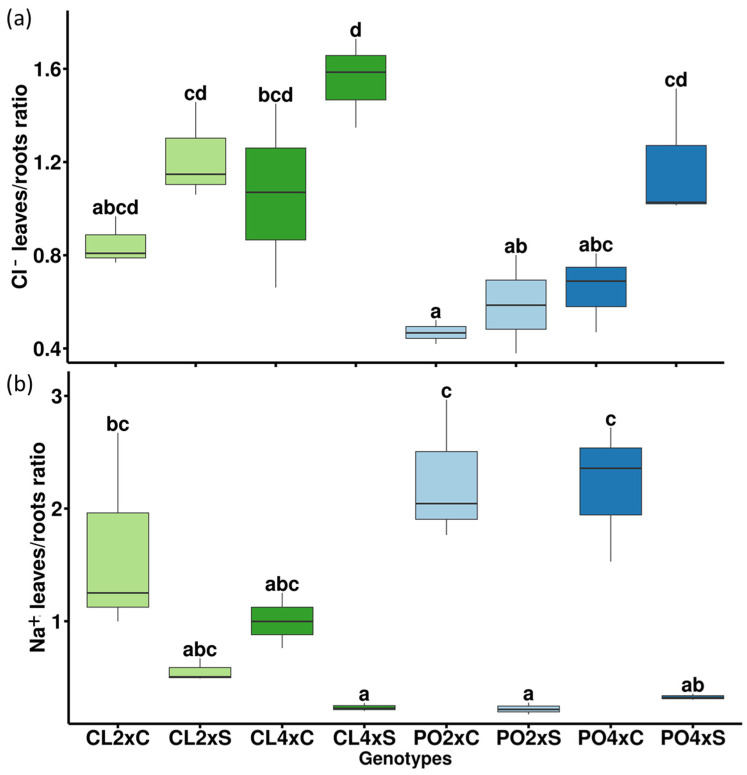
(**a**) Leaves/roots Cl^−^ ratio under control (C) and stress condition (S). (**b**) Leaves/roots Na^+^ ratio under control (C) and stress condition (S). Measurements were performed after four weeks of salt treatment (90 mM). Data represents 3 independent measurements (*n* = 3). Significance of the values were analyzed using Kruskal–Wallis test (*p* > 0.05) and mean comparison using Dunn’s test. Groups are separated by letters. Each group is assigned one or more letters. Groups sharing the same letter are not significantly different. Letter displays a clear and succinct way to present results of multiple comparisons.

**Figure 5:**
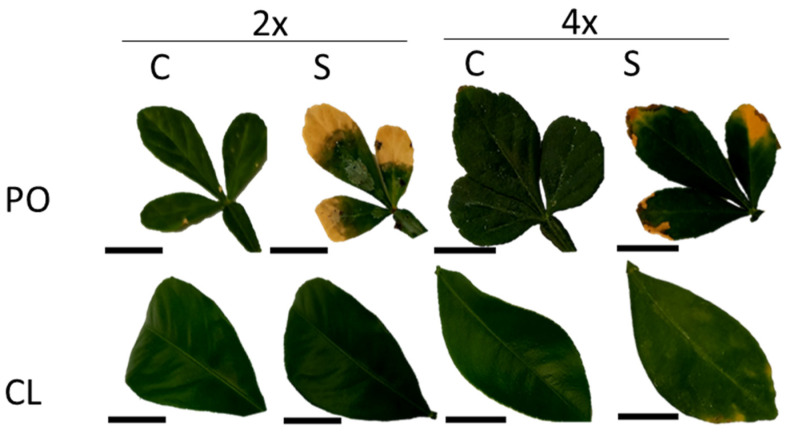
Leaves symptoms under Control (C) and Stressed (S) (W4 90 mM). Trifoliate orange (PO) Cleopatra mandarin (CL); Diploid (2x), tetraploid (4x). Bar represents 1 cm.

**Figure 6:**
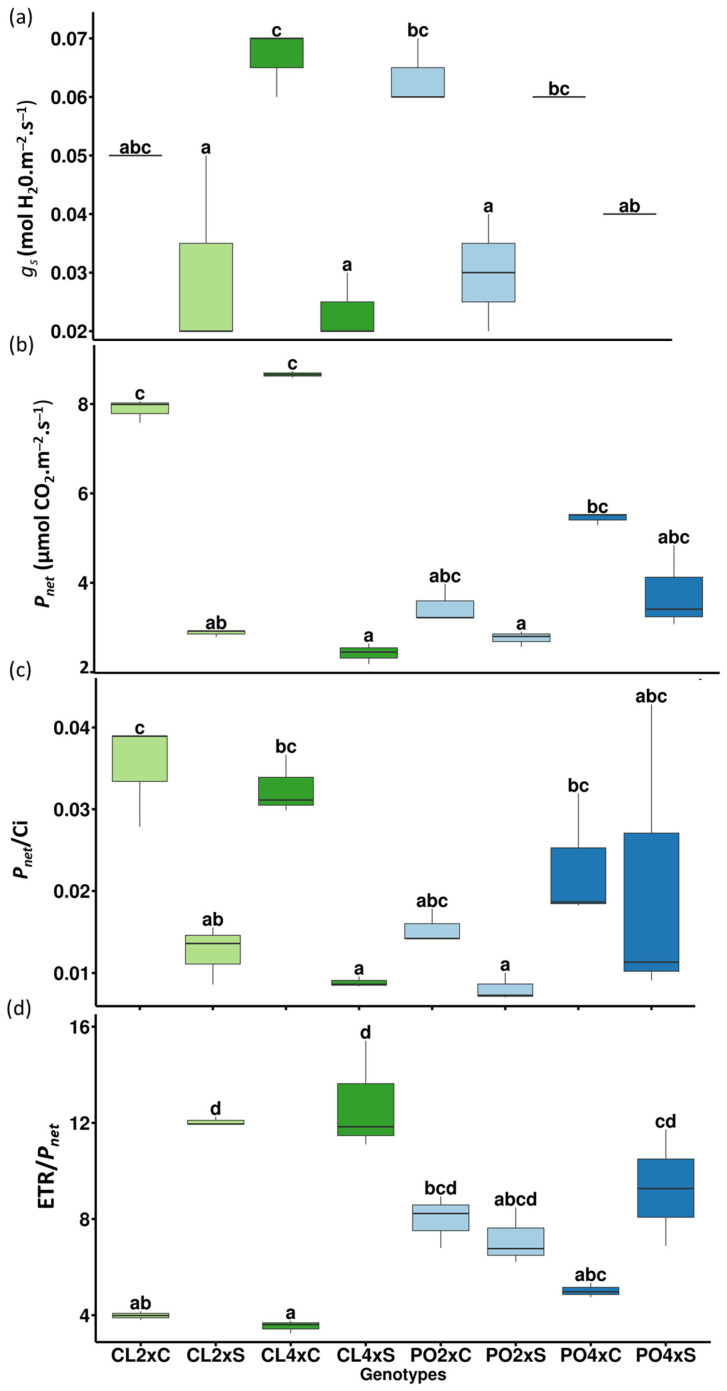
Photosynthetic performances under control (C) and salt stress conditions (S). Stomatal conductance (*g_s_*) (**a**), net photosynthesis (*P_net_*) (**b**), carboxylation efficiency (*P_net_*/ci) (**c**) and indicator of electron transport utilized by acceptors other than CO_2_ (ETR/*P_net_*) (**d**). Data represent 3 independent measurements (*n* = 3) after four weeks of salt treatment (90 mM). Significance of the values were analyzed using Kruskal–Wallis test (*p* > 0.05) and mean comparison using Dunn’s test. C and S represent Control and stressed plants, respectively. Groups are separated by letters. Each group is assigned one or more letters. Groups sharing the same letter are not significantly different. Letter displays a clear and succinct way to present results of multiple comparisons.

**Figure 7:**
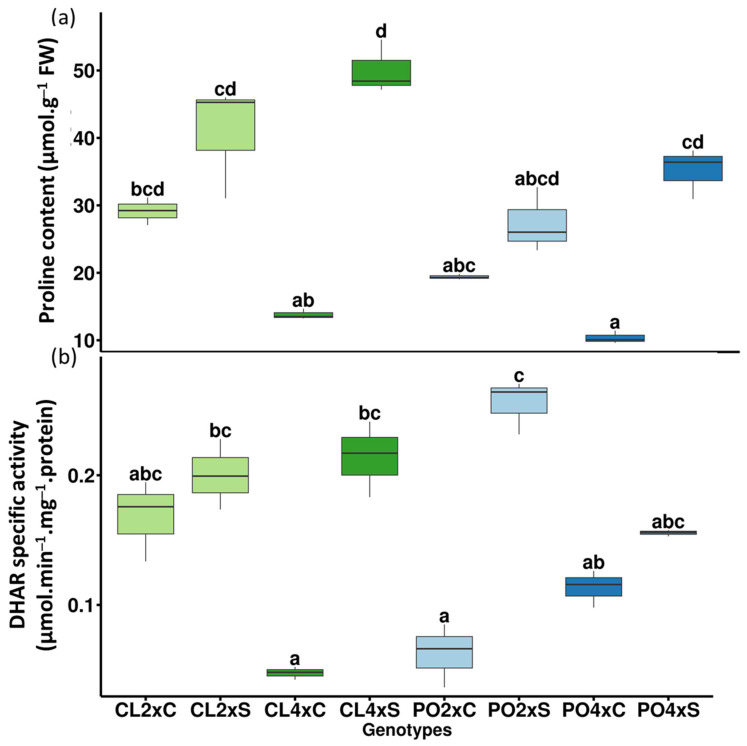
Ascorbate cycle, antioxidant metabolism and osmoprotection in leaves under control (C) and salt stress conditions (S). (**a**) Proline content and (**b**) DHAR specific activity. after four weeks of salt treatment (90 mM). Significance of the values were analyzed using Kruskal–Wallis test (*p* > 0.05) and mean comparison using Dunn’s test (*p*-value: < 0.05). C and S represent Control and stressed plants, respectively. Groups are separated by letters. Each group is assigned one or more letters. Groups sharing the same letter are not significantly different. Letter displays a clear and succinct way to present results of multiple comparisons.

In leaves (Table 3; Figure 2b), among the ten variables selected on PC1 and PC2, two were related to mineral uptake (K^+^ and Na^+^), four to photosynthetic performances (SPAD, gs, ETR, ETR/*P_net_*) and four to antioxidant metabolism (proline, APX, DHAR and MDA activities). Five variables explained the treatment effect (PC1), but unlike the roots, they were not related to mineral uptake. No apparent significant differences could be noticed for Na^+^ and Cl^−^ contents between control and stressed genotypes, except for PO4x Cl^−^ content (Figure 3c,d). Leaves/roots Cl^−^ and Na^+^ ratio under control and stressed conditions (Figure 4) was added to our analysis. This might be due to the short duration of the stress considered. The decrease in leaf greenness reported in Table 3 is concordant with leaf symptoms showed in Figure 5. Though not always significant, stomatal conductance (*g_s_*) decreased in all the genotypes tested (Figure 6a). Though not selected by sPLS-DA analysis, net photosynthesis (Pnet) and carboxylation efficiency (*P_net_*/Ci) decreased in most genotypes as an effect of salt stress (Figure 6b,c). In trend ETR/*P_net_*, values tended to increase in all the tested genotypes, except for the sensitive diploid trifoliate orange (PO2x) (Figure 6d). Significant increases in proline content and APX activity were observed between CL4xC and CL4xS and between PO4xC and PO4xS, respectively (Figure 7a,b, Table 3). Compared to the corresponding control, DHAR increased significantly for CL4x and PO2x, while *g_s_* decreased for both of them (Figure 6a and Figure 7b, Table 3). The five variables on PC2 (SPAD, ETR, Na^+^, K^+^ and MDA) discriminated significantly stressed PO2x from PO4x (Figure 2b). In a similar way, PO2xS had lower SPAD and MDA compared to the corresponding control (Figure 5, Table 3).

It is well known that plants cytosol contains 100–200 mM K^+^ and 1–10 mM Na^+^. For this reason, it is essential for plant cells to maintain a low cytosolic Na^+^ concentration and, therefore, a low Na^+^/K^+^ ratio in cells when under salt stress. We added the Na^+^/K^+^ calculation ratio in roots and leaves to give another inside on this question. The results showed, as expected, that Na^+^/K^+^ ratio in roots increased significantly in all the genotypes tested when comparing control and stressed genotypes (Figure S3). However, in leaves, only PO4x Na^+^/K^+^ ratio increased significantly. Because of the extreme sensitivity of PO2x to salt stress, we think that damages at leaves levels were too important to really conclude on the ability of PO2x to maintain its Na^+^/K^+^ ratio in leaves. However, strikingly, this result confirmed that CL genotypes strategy could cope with salt stress at the root level and preserve aerial part.

## 4. Discussion

Salt stress adversely affects physiological and biochemical processes associated with plant growth, development and yield. A set of responses at cellular, molecular, metabolic, physiological and whole-plant levels allows plants to cope with the negative effect of salinity. Tetraploidy usually enhances tolerance to various abiotic stresses in citrus [9]. To find out to what extent tetraploid citrus might have better adaptation capacities to salt stress than their corresponding diploids (2x), we monitored mineral uptake (seven parameters), photosynthesis performance indicators (thirteen parameters) and antioxidant metabolism (twelve parameters) in roots and leaves. Data were analyzed using multivariate analyses to reveal which are the most significant factors in explaining the differences in behavior of the plants tested.

Several physiological processes such as photosynthesis, respiration, starch metabolism and nitrogen fixation are affected under saline conditions. In citrus, salt stress tolerance is thought to be expressed through a wide genetic variability [1]. Tolerance traits are thought to be correlated with morphological (vigor), physiological (water use efficiency and chloride exclusion at the root level) [6] and biochemical (enhanced tolerance to oxidative stress) traits [24].

### 4.1. Photosynthetic Disturbances Reveal the Relative Sensibility of the Four Genotypes to Salt Stress

Among the tested parameters, the decrease in leaf greenness was the most obvious difference between the sensitive genotypes and the more tolerant ones. We evaluated the decrease in greenness in the citrus plants subjected to salt stress by monitoring SPAD measures along the stress period (Table 3; Figure 5). As expected, the decrease in SPAD values was only significant for the sensitive PO2x. Leaf greenness can be affected throughout different metabolic processes. By reducing water availability in the root zone, salt stress induces stomatal closure, which limits CO_2_ transfer into leaves and limits photosynthesis [25,26,27]. This limitation may decrease the activity of the Calvin–Benson cycle enzymes and lead to an over-reduction in the photosynthetic electron transport chain. When stress was applied, PLS-DA and sPLS-DA (Figure 2; Table 2) highlighted the importance of four variables related to this response, and linked to the photosynthetic metabolism (*gs*, *ETR*, *SPAD*, *ETR/P_net_*).

We noticed that in trend ETR/*P_net_*, values tended to increase in all the tested genotypes, except for the sensitive diploid trifoliate orange (PO2x) (Figure 6d). The increase in ETR/*P_ne_*_t_ ratio is known to represent an imbalance between the electron flow and the CO_2_ assimilation during photosynthesis. This disturbance is frequently associated with increases in oxygenase activity of Rubisco and might represent an electron flow to other physiological processes rather than to CO_2_ assimilation reactions [28,29]. Thus, it is admitted that the occurrence of increases in ETR/*P_net_* and decreases in *P_net_*/*Ci* might indicate a loss of photosynthetic efficiency in plants under salinity, especially in the ionic phase of salt stress [30]. However, an increase in ETR/*P_net_* ratio is not necessarily an indicator of sensitivity. To limit photo-inhibition, plants developed complex photo-protective mechanisms to dissipate excessive energy. In response to abiotic stresses, alternative electron sinks could be used in citrus plants [29,31]. Among this mechanisms, photochemical mechanisms (photorespiration and Mehler reaction) induced ROS production because of the use of O_2_ as alternative electron sink. However, NPQ (nonphotochemical chlorophyll fluorescence quenching) dissipate energy in excess as heat.

In another study on polyploidy citrus [18], it was observed that water deficit induced excess energy through photosystems in varieties as suggested by the correlation between ETR/*P_net_* and *P_net_*/*Ci*. Lourkisti et al. [18] observed that in 3× citrus, the electron flux through the thylakoid membrane could be maintained while the photosynthesis rate decreased, and excess energy induced by water deficit was effectively dissipated. In addition, they proposed that the great levels of NPQ associated with the positive correlation between NPQ and ETR/*P_net_* suggested that the thermal dissipation is the main photo-protective mechanism to eliminate the excess energy in 3x varieties. They also suggested that, inversely, the photo-protective mechanisms generating ROS production appeared to be less involved in preservation of photosystem apparatus in 3x varieties because of the negative correlation be-tween ETR/*P_net_* and CAT and SOD. In agreement with these authors, we suggest that all the genotypes tested have set up photo-protective mechanisms to limit water deficit-induced photo-inhibition. However, PO4x and CL genotypes’ better antioxidant capabilities could explain the absence of leaf symptoms.

Only CL4x showed a significant decrease in ETR. Stomatal conductance (*g_s_*), net photosynthesis (*P_net_*) and carboxylation efficiency (*P_net_*/*Ci*) decreased in most genotypes as an effect of salt stress (Figure 6a–c and Table 3). However, it is known that salt stress can cause electron chain destabilization and, more importantly, the disruption of carbon metabolism or phosphorylation [32]. Many studies reported that salt stress would decrease chlorophyll and carotenoid contents, potassium and magnesium uptake, photochemical efficiency, quantum yield of photosystem II and electron transport rates in photosystems I and II [33]. Stomatal closure reduces CO_2_ availability in leaves and inhibits carbon fixation [34,35]. Decreases in the rate of ATP and NADPH consumption for CO_2_ assimilation can lead to a decrease in the rate of linear electron transport rate (ETR). This is in agreement with previous results, suggesting that the salt stress tolerance capacity of Citrus seems to be strictly dependent on the capacity of the accession to reduce central metabolic processes related to carbon utilization and toxic ion exclusion [36].

As a result of salt stress, the efficiency of photosynthesis decreases and the generation of reactive oxygen species (ROS) such as superoxide (O_2_^−^), hydrogen peroxide (H_2_O_2_), hydroxyl radical (OH^−^) and singlet oxygen (^1^O_2_) increases [35,37,38,39]. All the genotypes tested suffered from a decrease in photosynthesis performances (Table 3; Figure 6). CL genotypes showed a significant decrease in *P_net_* and *P_net_/Ci* and a significant increase in *ETR/P_net_*. In PO genotypes, changes in photosynthetic performances were not significant, except for PO2x, which showed a significant decrease in leaf greenness SPAD values and stomatal conductance (*g_s_*) (Table 3). Similarly, Brumós et al. [36] reported that in comparison to CL mandarin, inhibition of photosynthesis, stomatal conductance and transpiration was lower (30% to 35%) in Carrizo citrange despite the considerable increase in foliar Cl^−^ content observed in the salinized leaves. This is believed to compensate for the reduced internal CO_2_ concentration in the leaf, preserving carbon assimilation under stress conditions. This allows maintenance of growth. Maintaining growth under salt stress conditions could be useful to dilute toxic ions in the shoot [36]. However, growth also requires a higher transpiration rate.

### 4.2. Ion Metabolism Is Differentially Affected by Salt Stress in the 4 Genotypes

In citrus, transpiration rate is directly related to Cl^−^ homeostasis. This explains why the Cl^−^-sensitive genotype such as trifoliate orange or Carrizo citrange had a higher Cl^−^ build-up [36]. In López-Climent et al. [40], the high-salinity-tolerant Citrus rootstock Foral-5 combined an efficient Cl^−^ exclusion mechanism with an active photosynthetic system, at elevated saline conditions, reinforcing the hypothesis that regulation of Cl^−^ homeostasis is a critical factor in determining NaCl tolerance in Citrus. When investigating mandarin accessions subjected to salt stress, Ben Yahmed et al. [12] demonstrated that mandarin accessions such as ‘Cleopatra’ and ‘Shekwasha’ presented few leaf symptoms and showed limited root-to-leaf Cl^−^ translocation. These authors also showed that mandarin accessions presenting the most severe leaf symptoms, such as ‘Fuzhu’, ‘Willowleaf’, ‘Beauty’, ‘King of Siam’ and ‘Nasnaran’, had the highest Cl^−^ translocation from root to leaf (leaf/root Cl^−^ ratio >1). Brumos et al. [36] also demonstrated that, in CL mandarin, salt stress did not lead to toxic chloride levels accumulated in the shoot. They suggested that this was the result of a more rapid root signaling. We also tested leaf/root Cl^−^ ratio and leaf/root Na^+^ ratio (Figure 4). Although differences between control and stressed genotypes were not significant in CL genotypes, the decrease in Na^+^ ratio was significant in PO genotypes. Conversely, CL genotypes showed the highest Cl^−^ ratio although differences between control and stressed genotypes were not significant. We noticed that the Na^+^ ratio tended to decrease in all the genotypes tested, while the Cl^−^ ratio increased between control and stressed conditions. This could possibly be explained by the low intensity of the stress considered (90 mM NaCl) and the short duration of the stress period (4 weeks).

Mineral elements, present as ions in plant cells, generally act as components of enzymes and coenzymes to regulate enzyme activity. They also play a crucial role in osmotic regulation and charge neutralization. Cell membrane stability and, hence, plant growth and development depend primarily on the balance of ionic metabolism. Salt stress induces an excessive accumulation of Na+ and Cl^−^ and a consequent deficiency of other vital ions, such as Ca^2+^ and K^+^ [39,41,42]. Increased Na^+^ content induces competitive inhibition of K^+^ (Na^+^ has a similar ionic radius and hydration energy to K^+^). Plant cells generally maintain a relatively high concentration of K^+^ and a relatively low concentration of Na^+^ in the cytoplasm to ensure their physiological activity. Consequently, excessive Na+ influx hampers K^+^ influx, leading to plant damage due to K^+^ deficiency. Ca^2+^ levels in cells are also reduced due to competitive Na^+^ inhibition. When the irrigation water loaded with solid salt is translocated from the roots to the aerial parts of the plant (translocation of Na^+^ and Cl^−^ ions into leaves (Figure 4)), the stress effect perceived by the plants corresponds to an ionic toxicity by sodium and/or chloride. Finally, if the stress is prolonged, salt stress leads to an unbalanced nutrition due to an interference with the absorption and transport of essential nutrients. Due to the interaction between Na^+^ and NH_4_^+^ and/or between Cl^−^ and NO_3_^−^, salinity contributes to reducing nitrogen (N) accumulation in plants. This, in turn, reduces growth and yield in salt-stressed plants [43]. Salinity may also lead to phosphorus (P) deficiency, as the ionic strength of PO_4_^3−^ and the low solubility of Ca-P minerals may reduce their activity. In our experiment, the stress applied was too short to observe mineral deficiency induced by salt stress. However, we did notice a significant difference in P content in CL2x and PO4x when comparing control and stressed conditions. Better ion storage capacity, and, thus, restricting transport of toxic ions in the root, may constitute an important characteristic explaining salt stress tolerance. Indeed, salinity-sensitive genotypes accumulate more Cl^−^ and Na^+^ in leaves while salinity-tolerant genotypes restrict Cl^−^ and Na^+^ in roots to limit chloride transport from roots to aerial parts and thus to better adapt to salinity [44]. Here, CL4x and PO4x tend to accumulate more Na^+^ and Cl^−^ ions in their roots than their diploid parents (Figure 3a,b). These different observations suggest that CL genotypes and PO4x could have a better capacity to restrict translocation and accumulate Cl^−^ ions in their roots. Ploidy could be the factor that enhances this capacity to tolerate salt stress more easily. Indeed, we noticed that in root samples, 13% of the variability were still detected with PC3, explaining ploidy level discrimination for both genotypes (Figure S2). The roots of 4x genotypes are generally thicker, less branched with less root development than those of 2x [45]. These histological characteristics could be associated with a lower hydraulic conductivity. Such an ability may be an advantage to cope with the effects of salt stress. Salt stress induces a very specific reaction in plants and is first perceived in the root zone.

After four weeks of salt treatment, the first component PC1 was able to discriminate the control versus the stressed samples, regardless of the genotype and ploidy level (Figure 2). PC2 clustered samples according to their genotypes and ploidy levels. In roots, PC3 explained ploidy level discrimination for both genotypes (Figure S2). This led us to wonder if we could identify phenotypic adaptations that lead to a better tolerance to salt stress or if better salt stress tolerance could be the consequence of constitutive differences.

Strikingly, in roots, eleven variables were selected by sPLS-DA analysis (Table 2) to discriminate control and stressed citrus tested. Five of these variables were linked to mineral uptake (Na^+^, Cl^−^, Ca^2+^, K^+^/Na^+^, P). As expected, Cl^−^ and Na^+^ content increased in most stressed genotypes. However, the increase in Na^+^ content was significantly different when comparing Cl4xC and Cl4xS and PO4xC and PO4xS, respectively (Figure 3a,b), contrary to their 2x counterparts. An increase in Cl^−^ was only significant in PO4x leaves when comparing control and stressed plant (Figure 3a,b). However, contrary to PO2x and PO4x, Ca^2+^ leaves content decreased when salt stress was applied in CL4x genotypes, while it increased in PO genotype (Table 3). Mineral elements present as ions in plant cells usually act as components of enzymes and coenzymes to regulate enzyme activity. They also play a crucial role in osmotic regulation and charge neutralization. Stability of the cell membrane, and, therefore, plant growth and development, depends mostly on the balance of ion metabolism. Ca^2+^ concentration decreased, as expected, in CL4x roots. Our results could be explained by two different strategies of response to salt stress. Indeed, regulation of osmotic pressure in CL, due to Na^+^ concentrations, could be partly achieved through the compensatory decrease in Ca^2+^. As an important component of signal transduction, Ca^2+^ channels are induced to open when plants are subjected to salt stress. Released from the vacuole, Ca^2+^ binds with calmodulin or other calcic binding proteins and, thereby, regulates cell metabolism and gene expression, promoting plant stress response. Increasing concentration of Ca^2+^ in PO could be the reflection of a signaling pathway activation. Moreover, in CL, increasing concentration of inorganic ions such as K^+^, and organic substances like proline and glycine betaine under salt stress, could also be a way to maintain plant’s ability to absorb water from the environment [44]. We observed a significant increase in proline content in CL root, contrary to PO (Table 3). PO2x strategy seems limited, as this genotype recorded the most important decrease in leaf greenness in our experimentation (Table 3). We also observed a significant decrease in the potassium K^+^/Na^+^ ratio in stressed plants compared to controls (Table 3), which could reflect the disturbance in plant growth and development. Growth disturbance under salt stress could also be the result of the interaction between Na^+^ and NH_4_^+^ and/or between Cl^−^ and NO_3_^−^. This contributes to reduced accumulation of nitrogen (N) in plants and could lead to a deficiency of phosphorus (P) because of the ionic strength that could decrease the activity of PO_4_^3−^, and the low solubility of Ca-P minerals. Due to the short duration of stress, P deficiency cannot be observed in our results, where P variation between control and stressed conditions were not significant, except for PO4x (increase of 1,6-fold).

### 4.3. Could a Better Osmoregulation and a Better Antioxidant Capacity Explain Better Tolerance to Salt Stress?

High osmotic stress is the first effect perceived in the early beginning of salt stress, due to a low external water potential. Among the phenomena involved in the response to salt stress, many publications demonstrated that proline biosynthesis and/or accumulation allow osmoprotection [46,47,48]. Proline not only provides a means to cope with osmotic stress, it can act as an organic nitrogen reserve during stress recovery, acts as an antioxidant (ROS scavenger) and as a ^1^O_2_ quencher to protect the photosynthetic apparatus. Thus, proline is a central component of plant adaptation against salt stress [49,50]. In our experiment, higher proline content was recorded in the leaves of 4x PO and CL genotypes (Table 3 and Figure 7a). Proline content increased significantly in CL2x roots, while this increase was not significant in leaves (Table 3 and Figure 7a). However, in roots of CL4x, proline increase was significant (Table 3). As an organic solute, better proline accumulation could mean better ability to fight against osmotic pressure induced by salt stress. In that way, Cleopatra genotypes, especially 4x genotypes, could have better capacities to fight against salt stress.

As oxidation indicator, MDA content allow us to identify which genotype could be the most affected by salt stress. When salt stress was applied, PO2xS genotype showed the highest level of MDA under stress conditions in leaves (Table 3) and the greatest decrease in leaf greenness (Table 3 and Figure 5). Oxidative stress damage can be estimated by assaying the products of ROS detoxification such as O_2_^●−^ [24]. The removal of the O_2_^●−^ radical occurs in two steps. First, superoxide dismutase (SOD), converts the O_2_^●−^ radical to hydrogen peroxide (H_2_O_2_) [51]. In another study, increased specific SOD activity found in 3x genotypes and Ellendale tangor was related to low MDA levels and, thus, the limitation of the lipid peroxidation process [52]. The H_2_O_2_ formed by SOD can then be converted to oxygen and water by catalase. Alternatively, it can also diffuse rapidly across membranes via aquaporins to other compartments and join the ascorbate-glutathione cycle. The oxidation of glutathione and reduction in ascorbate allow the removal of H_2_O_2_ [53]. APX and DHAR are part of the glutathione-ascorbate cycle, which plays a crucial role in the ROS-detoxification process. In the first reaction catalyzed by ascorbate peroxidase (APX), ascorbate acts as a reducing agent and oxidizes to monodehydroascorbate [54,55]. In their study, Lourkisti et al. (2022) [52] reported that increased APX and CAT activities in most of the 3x citrus genotypes appeared to be sufficient to prevent oxidative damage by reducing H_2_O_2_ accumulation.

**Figure 8:**
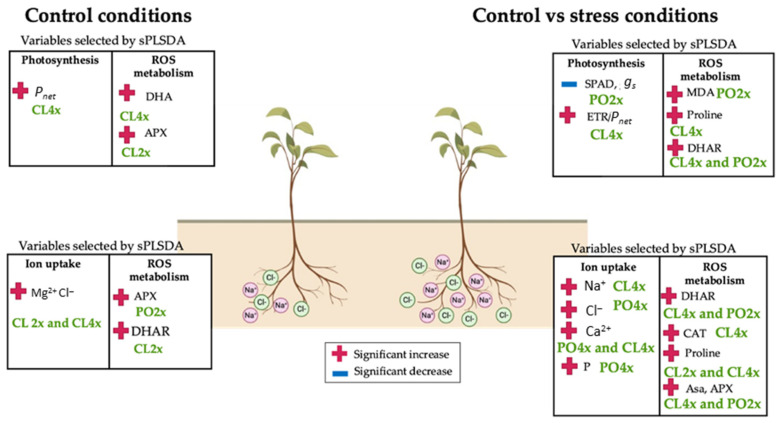
Summary: biological significance of principal variables selected by sPLS-DA in leaves and roots of Trifoliate orange (PO) and Cleopatra (CL) genotypes under control and stress conditions. This figure design was realized using Biorender (biorender.com).

In our experiment, APX activity was significantly different when comparing Cl4xC and Cl4xS and PO4xC and PO4xS, respectively. This result suggests that in 4x genotype, the ascorbate-glutathione cycle could be one of the pathways involved to limit ROS accumulation. We also found that DHAR increased significantly between control and stressed for CL4x and PO2x. Although this variable was not selected by sPLS-DA, we found that Asa/DHA ratio was also significantly different in CL4x and PO2x when comparing control and stressed plant at the root level (Figure S3). This result suggests that, in CL4x, at the root level, DHAR activity was enough for an effective recycling of Asa and contributed to stronger antioxidant capacity. However, in PO2x, the increase in DHAR activity in response to osmotic choc in roots which impaired recycling process of Asa in these genotypes could explain their lack of tolerance. Antioxidant performances of the PO genotypes were not as strong as what was observed in the CL genotype. Indeed, for example, PO2x showed no significant increase in roots and leaves proline content. However, PO4x as CL4x showed a significant increase in the proline content in leaves (Table 3). This increase is also significant in roots for CL4x only. CL4x was the only genotype where roots Asa, CAT, APX and DHAR activity increased significantly at the same time. Taken together, these results suggest that CL genotype could have better antioxidant capacities. This better antioxidant capacity, in addition to better osmoprotectant synthesis and ion homeostasis, could explain that CL mandarin stress response seems to be more efficient in coping with salt stress. However, tetraploidy improved salt stress tolerance in PO and CL genotypes (Figure 8).

## 5. Conclusions

Under salt stress conditions, all the tested genotypes showed a different response to salinity. These differences allowed, firstly, to identify PO2x as the most sensitive genotype. Secondly, they allowed us to better understand the response of the most tolerant genotypes, CL4x and PO4x. According to our results, the most salt-stress-tolerant genotypes could maintain (1) their photosynthetic machinery, (2) a strong antioxidant defense mechanism, (3) a capacity for storage, transport and absorption of minerals and nutrients. Overall, our work demonstrated that CL genotypes could have a better adaptive capacity than the PO ones, especially the CL4x, even if tetraploidy had enhanced tolerance of the sensitive trifoliate orange. To summarize, our results could lead to a classification of the genotypes studied in these experiments based on their salt tolerance capacities. Starting from the most sensitive genotype to the most tolerant, genotypes tend to be ordered as follows: PO2x, PO4x, CL2x and CL4x.

To deepen our understanding of the molecular mechanism involved in the stress response, a transcriptomic study will be further performed. It will further highlight different pathways that regulate the complex process of salt stress adaptation. This integrative study will help the researchers to design effective strategies to fight against salt stress.

## Figures and Tables

**Table 1:** Plant material.

Common Name	Scientific Name	Status	Ploidy Level	ICVN/SRA
*Cleopatra mandarin*	*C. reshni*	Parent	2x	ICVN-0110274
*Trifoliate Orange*	*P. trifoliata*	Parent	2x	ICVN-0110081
*Cleopatra mandarin*	*C. reshni*	doubled diploid	4x	ICVN-0101110
*Trifoliate Orange*	*P. trifoliata*	doubled diploid	4x	ICVN-01011106

**Table 2:** Variable selection by sPLS-DA for control and stressed leaves and roots: X and Y are coefficient of regression for each principal component (PC).

Root Variables	X (PC1)	Y(PC2)
Na^+^	−0.87	0.27
K^+^/Na^+^	0.84	−0.27
P	−0.20	0.85
Cl^−^	0.00	0.90
Ca^2+^	0.00	0.86
CAT	−0.81	0.34
Proline	−0.83	−0.30
**Leaf Variables**	**X (PC1)**	**Y (PC2)**
K^+^	0.25	−0.75
Na^+^	−0.09	0.86
ETR	0.21	0.56
SPAD	0.93	−0.10
ETR/*P_net_*	−0.76	−0.13
gs	−0.90	0.21
Proline	−0.88	0.24
MDA	−0.92	0.08
DHAR	0.81	−0.42
APX	−0.92	0.08

Chloride (Cl^−^), calcium (Ca^2+^), and potassium (K^+^) content were expressed in mg·g^−1^. DHAR, APX and CAT activities were expressed in μmol·min^−1^·mg^−1^·protein. Proline was expressed in μmol·g^−1^. MDA was expressed in nmol·g^−1^ FW. *g_s_* was expressed in mol H_2_O·m^−2^·s^−1^. Chlorophyll content (SPAD) was expressed in SPAD units.

**Table 3:** Statistical analysis of biochemical and photosynthetic variables selected using sPLS-DA, for trifoliate orange and Cleopatra, 2x and 4x, in control (C) and stressed (S) leaves and roots.

Pairwise Comparison											
Tissu	Variable	Pvalue	Size Effect	CL2xC	CL2xS	CL4xC	CL4xS	PO2xC	PO2xS	PO4xC	PO4xS
Roots	Na^+^	3.44 × 10^−3^	0.89	4.68 ^ab^	10.76 ^bcd^	6.71 ^abc^	21.30 ^d^	2.39 ^ab^	12.79 ^bcd^	2.03 ^a^	18.37 ^cd^
	Cl^−^	3.22 × 10^−3^	0.90	50.63 ^abc^	68.44 ^bc^	53.96 ^abc^	85.26 ^c^	22.42 ^a^	39.93 ^ab^	26.39 ^a^	76.58 ^c^
	Ca^2+^	4.30 × 10^−3^	0.86	10.92 ^abcd^	6.67 ^ab^	11.36 ^bcd^	6.39 ^a^	9.12 ^abc^	12.37 ^cd^	7.01 ^ab^	15.42 ^d^
	K^+^/Na^+^	2.89 × 10^−3^	0.91	5.33 ^cde^	1.12 ^ab^	4.29 ^bcde^	0.85 ^a^	13.17 ^e^	2.54 ^abcd^	9.34 ^de^	2.25 ^abc^
	P	1.03 × 10^−3^	0.71	3.32 ^abcd^	2.05 ^a^	2.56 ^bcd^	2.52 ^ab^	3.01 ^abc^	4.70 ^cd^	2.89 ^ab^	5.14 ^d^
	DHAR	7.39 × 10^−3^	0.77	0.21 ^cd^	0.20 ^bcd^	0.01 ^a^	0.21 ^cd^	0.02 ^ab^	0.29 ^d^	0.15 ^abc^	0.20 ^bcd^
	CAT	6.01 × 10^−3^	0.80	1.57 ^ab^	2.90 ^abc^	1.15 ^a^	3.49 ^bc^	0.88 ^a^	3.79 ^bc^	1.26 ^ab^	5.52 ^c^
	Proline	8.88 × 10^−3^	0.74	22.92 ^a^	33.93 ^bc^	26.31 ^ab^	44.72 ^c^	16.57 ^a^	26.67 ^abc^	24.98 ^ab^	26.57 ^abc^
	MDA	6.01 × 10^−2^	0.41	3.14 ^ab^	4.43 ^bc^	1.58 ^a^	4.63 ^bc^	3.57 ^abc^	6.62 ^c^	3.12 ^ab^	4.33 ^bc^
	Asa	1.61 × 10^−2^	0.64	3.21 ^ab^	3.99 ^b^	1.87 ^a^	4.53 ^b^	1.73 ^a^	5.15 ^b^	3.11 ^ab^	3.41 ^ab^
	APX	1.61 × 10^−2^	0.64	0.47 ^cd^	0.46 ^bcd^	0.02 ^a^	0.49 ^cd^	0.05 ^ab^	0.67 ^d^	0.35 ^abc^	0.45 ^bcd^
Leaves	Na^+^	2.35 × 10^−3^	0.57	6.37 ^c^	5.77 ^bc^	6.48 ^c^	5.07 ^abc^	5.15 ^abc^	2.81 ^a^	4.33 ^ab^	5.99 ^bc^
	K^+^	6.34 × 10^−3^	0.73	16.39 ^ab^	19.06 ^abcd^	17.79 ^abc^	14.62 ^a^	23.88 ^bcd^	28.54 ^d^	28.34 ^cd^	15.60 ^a^
	ETR	5.36 × 10^−3^	0.81	31.37 ^bcd^	34.67 ^d^	32.73 ^cd^	26.20 ^ab^	27.43 ^abc^	19.63 ^a^	27.33 ^abc^	33.63 ^cd^
	SPAD	2.77 × 10^−3^	0.92	71.9 ^abc^	58.57 ^ab^	69.33 ^ab^	72.80 ^bc^	76.20 ^bc^	38.03 ^a^	80.57 ^c^	80.33 ^c^
	gs	4.94 × 10^−3^	0.83	0.05 ^abc^	0.03 ^a^	0.07 ^c^	0.02 ^a^	0.06 ^bc^	0.03 ^a^	0.06 ^bc^	0.04 ^ab^
	ETR/*P_net_*	2.90 × 10^−3^	0.92	3.98 ^ab^	12.05 ^d^	3.54 ^a^	12.79 ^d^	7.99 ^bcd^	7.16 ^abcd^	5.02 ^abc^	9.29 ^cd^
	MDA	9.78 × 10^−3^	0.72	5.97 ^abc^	10.78 ^bc^	4.99 ^ab^	4.99 ^ab^	1.55 ^a^	29.54 ^c^	3.81 ^a^	5.97 ^abc^
	Proline	3.22 × 10^−3^	0.90	29.15 ^bcd^	40.78 ^cd^	13.81 ^ab^	50.05 ^d^	19.40 ^abc^	27.36 ^abcd^	10.37 ^a^	35.15 ^cd^
	DHAR	2.90 × 10^−3^	0.92	0.17 ^abc^	0.20 ^bc^	0.05 ^a^	0.21 ^bc^	0.06 ^a^	0.26 ^c^	0.11 ^ab^	0.16 ^abc^
	APX	2.90 × 10^−3^	0.92	0.38 ^abc^	0.46 ^bc^	0.11 ^a^	0.49 ^bc^	0.26 ^ab^	0.35 ^abc^	0.14 ^a^	0.58 ^c^

Chloride (Cl^−^), calcium (Ca^2+^), and potassium (K^+^) contents were expressed in mg·g^−1^. DHAR, APX and CAT activities were expressed in μmol·min^−1^·mg^−1^·protein. Asa and Proline were expressed in μmol·g^−1^. MDA was expressed in nmol·g^−1^ FW. *g_s_* was expressed in mol H_2_O·m^−2^·s^−1^. Chlorphyll content (SPAD) was expressed in SPAD units. Non-parametric Kruskal–Wallis test was applied (*p* ≤ α, with α = 0.05)) as well as the size effect Eta2 which defined the magnitude of the difference between 2 groups (0.2 = small, 0.5 = medium, 0.8 = large). Mean comparison by Dunn’s test was performed at *p*-value < 0.05. Groups sharing the same letter are not significantly different. Letter displays a clear and succinct way to present results of multiple comparisons.

## Data Availability

The original contributions presented in this paper are included in the article and in the Supplemental Data. Further inquiries can be directed to the corresponding author/s.

## Supplementary Materials

The following supporting information can be downloaded at: https://www.mdpi.com/article/10.3390/antiox12081640/s1, Table S1: Coefficient of regression (ncomp = 2) for variables selected by sPLS-DA for control leaves and roots. Table S2: Statistical analysis of biochemical and photosynthetic variables selected using sPLS-DA, for 2x and 4x Trifoliate orange and Cleopatra in control leaves and roots. Significance of variation, size effect, and pairwise comparison between groups. Table S3: Variable selection by sPLS-DA for control and stressed leaves and roots: X and Y are coefficient of regression for each principal component (PC). Figure S1: Biplot of PLS-DA for control (C) and stressed (S) roots on PC2 and 3. Measurements were performed after four weeks of salt treatment (90 mM). Figure S2: Root Asa/DHA under control (C) and salt stress conditions (S) after four weeks of salt treatment (90 mM). Significance of the values were analyzed using the Kruskal–Wallis test (*p* > 0.05) and mean comparison using Dunn’s test (*p*-value < 0.05). C and S represent Control and stressed plants, respectively. AsA and DHA were expressed in µmol·g^−1^. Groups are separated by letters. each group is assigned one or more letters. Groups sharing the same letter are not significantly different. Letter displays a clear and succinct way to present results of multiple comparisons. Figure S3: root Na^+^/K^+^ ration under control (C) and salt stress conditions (S) after four weeks of salt treatment (90 mM) in roots (a) and in leaves (b). Significance of the values were analyzed using Kruskal–Wallis test (*p* > 0.05) and mean comparison using Dunn’s test (*p*-value < 0.05). C and S represent Control and stressed plants, respectively. Na+ and K+ content were expressed in mg/g. Groups are separated by letters. each group is assigned one or more letters. Groups sharing the same letter are not significantly different. Letter displays a clear and succinct way to present results of multiple comparisons.
