# Supplementary material for: Insight into Physiological and Biochemical Determinants of Salt Stress Tolerance in Tetraploid Citrus
Source: Antioxidants (Basel). 2023 Aug 19;12(8):1640. doi: 10.3390/antiox12081640 (PMC10451669; doi:10.3390/antiox12081640)
Supplement: Supplementary file 1 [file antioxidants-12-01640-s001.zip › antioxidants-2552627-supplementary.pdf]

antioxidants MDPI

Supplemental

Table S1. Coefficient of regression (ncomp = 2) for variables selected by sPLS-DA for control leaves and roots.

Tissu	Leaf variable	X (PC1)	Y (PC2)
Roots	Cl^-	-1.00	0.00
	Mg^{2+}	0.97	-0.08
	DHAR	-0.18	-0.99
	APX	-0.18	-0.99
Leaves	P_{net}	-0.99	0.09
	DHA	-0.95	0.14
	CAT	0.35	-0.87
	APX	-0.36	-0.93

Chloride (Cl^-) and Magnesium (Mg^{2+}) content were expressed in mg.g^{-1}. DHAR, APX and CAT activity were expressed in µmol. min^{-1}. mg^{-1} protein. DHA was expressed in µmol.g^{-1}. P_{net} was expressed in µmol. m^{-1}.s^{-1}.

Table S2. Statistical analysis of biochemical and photosynthetic variables selected using sPLSDA, for 2x and 4x Trifoliate orange and Cleopatra in control leaves and roots. Significance of variation, size effect, and pairwise comparison between groups.

				Pairwise comparison			
**Tissu**	**Variable**	**Pvalue**	**Size effect**	**CL2x**	**CL4x**	**PO2x**	**PO4x**
Control roots	Cl^-	0.0361	0.692	50.63^{bc}	53.96^{c}	22.42^{a}	26.39^{ab}
	Mg^{2+}	0.032	0.726	5.67^{b}	6.08^{b}	3.24^{ab}	2.76^{a}
	DHAR	0.0286	0.756	0.21^{b}	0.01^{a}	0.15^{b}	0.02^{ab}
	APX	0.0286	0.756	0.47^{b}	0.02^{a}	0.35^{b}	0.05^{ab}
Control leaves	P_{net}	0.0150	0.932	7.88^{bc}	8.66^{c}	3.47^{a}	5.44^{ab}
	DHA	0.0345	0.705	31.31^{ab}	39.58^{b}	1.43^{a}	1.53^{a}
	CAT	0.0213	0.838	3.03^{b}	0.78^{a}	1.89^{ab}	2.68^{b}
	APX	0.0156	0.923	0.38^{b}	0.11^{a}	0.14^{a}	0.26^{ab}

Chloride (Cl^-) and Magnesium (Mg^{2+}) content were expressed in mg.g^{-1}. DHAR, APX and CAT activity were expressed in µmol. min^{-1}. mg^{-1} protein. DHA was expressed in µmol.g^{-1}. P_{net} was expressed in µmol. m^{-1}.s^{-1}. Non-parametric Kruskal-Wallis test was applied ($p \leq \alpha$, with $\alpha = 0.05$)) as well as the size effect Eta2 which defined the magnitude of the difference between 2 groups (0.2=small, 0.5=medium, 0.8=large). Pairwise comparison by Dunn's test was performed at p-value < 0.05. Groups are separated by letters. each group is assigned one or more letters. Groups sharing the same letter are not significantly different. Letter displays a clear and succinct way to present results of multiple comparisons.

Table S3. Variable selection by sPLS-DA for control and stressed leaves and roots: X and Y are coefficient of regression for each principal component (PC).

Root variables	X (PC2)	Y(PC3)
Ca^{2+}	-0.87	0.27
P	0.84	-0.27
MDA	-0.92	-0.16
Asa	-0.76	0.00
DHAR	-0.64	-0.06
APX	-0.80	-0.16

Calcium (Cl^-) and potassium (Mg^{2+}) content were expressed in mg.g^{-1}. DHAR and APX activity were expressed in µmol. min^{-1}. mg^{-1} protein. AsA was expressed in µmol.g^{-1}. P_{net} was expressed in µmol. m^{-1}.s^{-1}.

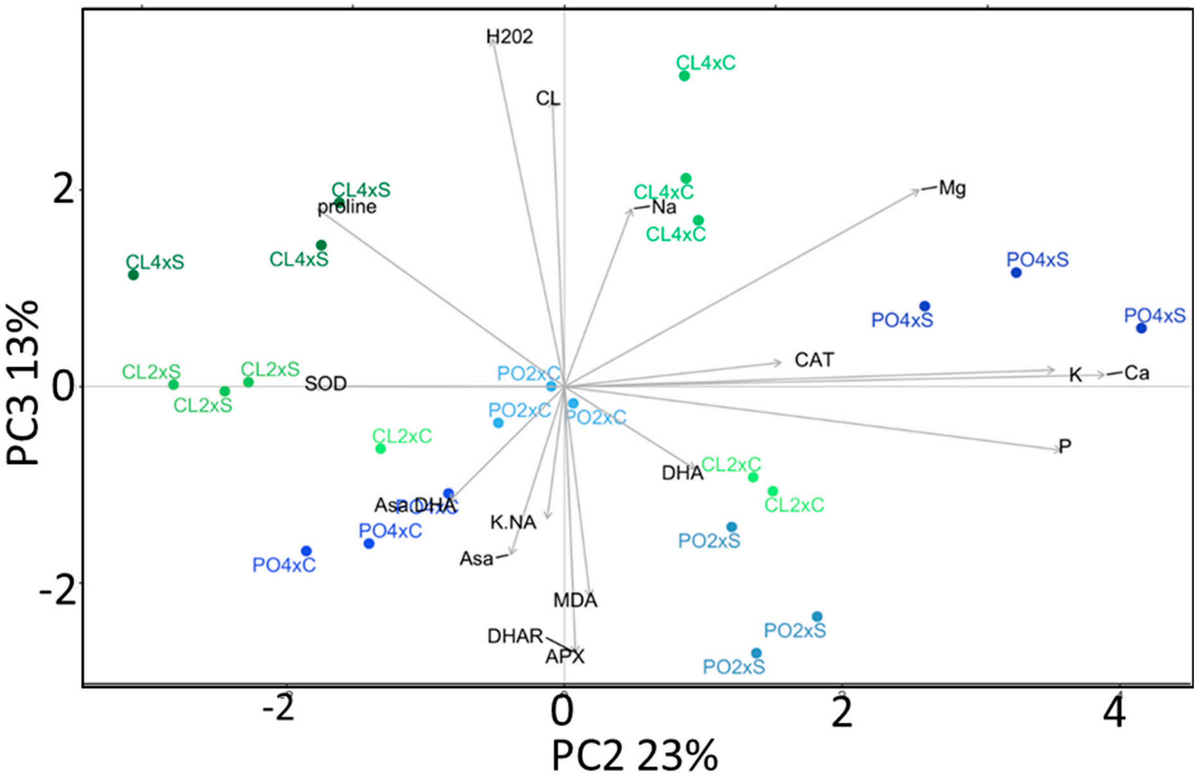

Figure S1. Biplot of PLS-DA for control (C) and stressed (S) roots on PC2 and 3. Measurements were performed after four weeks of salt treatment (90 mM).

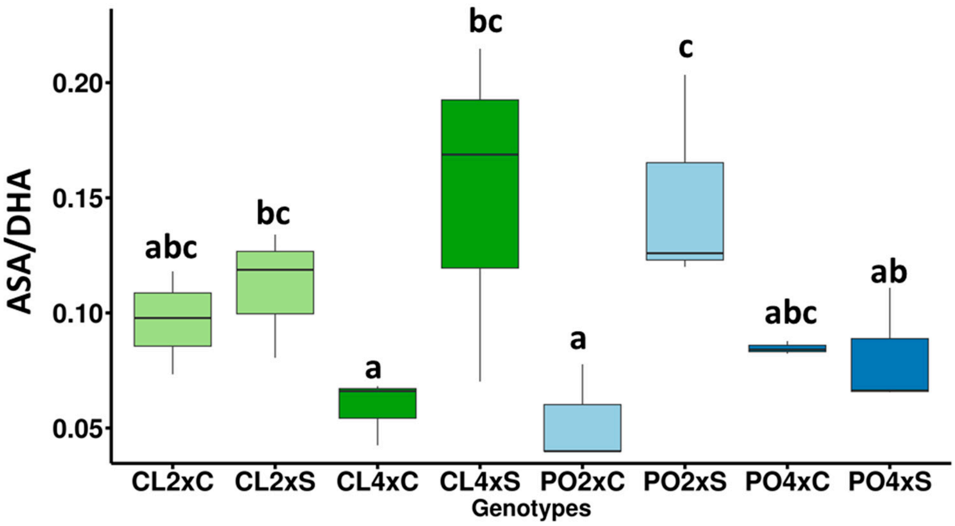

Figure S2. Root Asa/DHA under control (C) and salt stress conditions (S) after four weeks of salt tratment(90 mM). Significance of the values were analyzed using Kruskal wallis test (P>0.05) and mean comparison using Dunn test (pval<0.05).C and S represent Control and stressed plants respectively. AsA and DHA were expressed in mol.g-1. Groups are separated by letters. each group is assigned one or more letters. Groups sharing the same letter are not significantly different. Letter displays a clear and succinct way to present results of multiple comparisons.

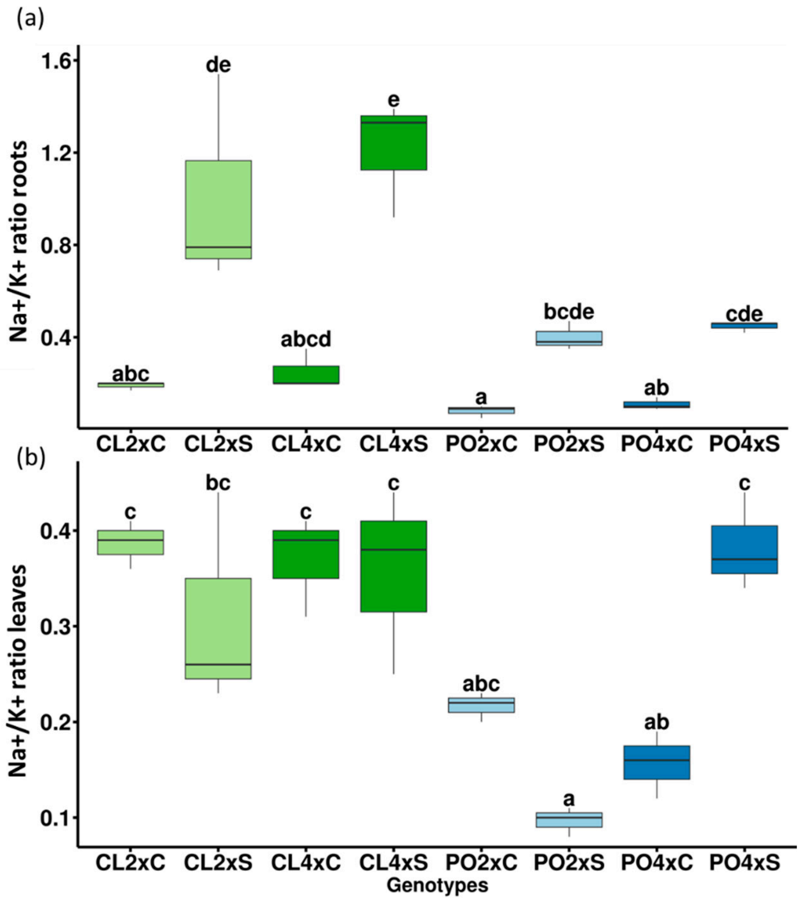

Figure S3. root Na+/K+ ration under control (C) and salt stress conditions (S) after four weeks of salt tratment(90 mM) in roots (a) and in leaves (b). Significance of the values were analyzed using Kruskal wallis test (P>0.05) and mean comparison using Dunn test (pval<0.05).C and S represent Control and stressed plants respectively. Na+ and K+ content were expressed in mg/g. Groups are separated by letters. each group is assigned one or more letters. Groups sharing the same letter are not significantly different. Letter displays a clear and succinct way to present results of multiple comparisons.
